# Comparison of an ensemble machine learning model to a Cox regression model to predict colorectal cancer risk among people with HIV using retrospective nationwide cohort data in Sweden: a study protocol

**DOI:** 10.1136/bmjopen-2025-108693

**Published:** 2026-09-09

**Authors:** Josefin Nilsson, Yunyang Deng, Olof Elvstam, Isabela Killander-Möller, Jiayao Lei, Fredrik Mansson, Pontus Naucler, Jonas Nygren, Suzanne Ruhe-van der Werff, Philippe Wagner, Aylin Yilmaz, Johanna Brännström, Magnus Boman, Christina Carlander

**Affiliations:** 1 Unit of Infectious Diseases and Dermatology, Department of Medicine Huddinge, Karolinska Institute, Huddinge, Sweden; 2 Department of Medical Epidemiology and Biostatistics, Karolinska Institute, Stockholm, Sweden; 3 Department of Translational Medicine, Lund University, Malmö, Sweden; 4 Department of Infectious Diseases, Växjö Central Hospital, Växjö, Sweden; 5 Department of Clinical Sciences, Lund University, Infectious Diseases Research Unit, Malmo, Sweden; 6 Department of Infectious Diseases, Karolinska University Hospital, Stockholm, Sweden; 7 Department of Clinical Sciences, Danderyd Hospital, Karolinska Institute, Stockholm, Sweden; 8 Department of Surgery, Ersta Hospital, Stockholm, Sweden; 9 Uppsala University, Uppsala, Sweden; 10 Department of Infectious Diseases, University of Gothenburg, Gothenburg,Sweden; 11 Department of Infectious Diseases, Sahlgrenska University Hospital, Gothenburg, Sweden; 12 Department of Clinical Science and Education, Södersjukhuset, Stockholm, Sweden; 13 Department of Infectious Diseases, Södersjukhuset, Stockholm, Sweden; 14 Department of Medicine Solna, Karolinska Institute, Stockholm, Sweden

**Keywords:** Machine Learning, STATISTICS & RESEARCH METHODS, HIV & AIDS, Epidemiology, Methods, EPIDEMIOLOGY

## Abstract

**Abstract:**

**Introduction:**

There are currently no colorectal cancer (CRC) screening recommendations specifically outlined for people with HIV (PWH). Screening measures used for people without HIV (PWoH) have been previously discussed as sufficient for use among PWH, despite observations of higher CRC prevalence and CRC reportedly appearing at earlier ages among PWH in comparison to PWoH. Machine learning (ML) methods are regarded as robust approaches that may enhance predictive performance, particularly in the context of complex or high-dimensional data. This study aims to develop an ensemble ML model to predict CRC risk in PWH using comprehensive nationwide datasets. The model’s predictive performance will be evaluated and compared with a baseline Cox proportional regression model. The better-performing method will be implemented to develop a CRC risk prediction model with the aim of personalising screening recommendations for PWH.

**Methods and analysis:**

The study population will include all PWH and PWoH born between 1940 and 2008, aged 18 or older and living in Sweden sometime between 1983 and 2024. The study population will be linked to six nationwide demographic and healthcare registers. Follow-up will continue until the first incident of CRC, emigration or death. The outcome of interest is CRC. PWH will be matched to negative controls 1:10. A Cox regression analysis will be completed first, and the results will be used as a baseline comparison to the ensemble ML results. A range of ML methods will be used to develop the ensemble model using stacking.

**Ethics and dissemination:**

This study has ethical approval from the Regional Ethical Committee in Sweden (Dnr: 2024-04185-02, 2024-06783-02, 2023-00191-01, 2022-02897-02, 2022-05624-01, 2018/11-31/2). Given that the study is retrospective and register-based, using only pseudonymised data, there are minimal physical, psychological or privacy risks to included individuals. All results will be presented at the population level with no possibility of identification. The results of this study will be submitted for publication in a peer-reviewed journal.

**STRENGTHS AND LIMITATIONS OF THIS STUDY**

- This study will include comprehensive data coverage with >99% of people with diagnosed HIV in Sweden spanning over 30 years of detailed follow-up through high-quality national registers.
- There has been strong patient involvement through a diverse reference group, ensuring alignment with community needs.
- The study will include all people with HIV in Sweden and therefore the results will be nationally generalisable, as well as potentially to external populations with similar demographics and treatment availability, such as other European countries and Canada.
- While the integration of multiple machine learning techniques within a robust and comprehensive methodological framework may improve prediction performance, it may reduce model interpretability.
- The retrospective study design restricts the analyses to preselected variables, limiting the inclusion of any potentially important or confounding factors identified during the course of the study, which could increase the risk of remaining residual confounding.

## Introduction

 Adverse non-AIDS health outcomes are more prevalent among people with HIV (PWH) in comparison to people without HIV (PWoH), including increased incidence of mortality from non-AIDS-related cancers.^1^ Notably, an increased risk of cancers has been observed among individuals who are on antiretroviral therapy (ART) and virally suppressed.^1^ Prior studies have indicated that this increased risk is probably multifactorial, including risk factors such as HIV-related chronic inflammation, immunosuppression and ART toxicity, but also lifestyle factors.^2 3^

Colorectal cancer (CRC) has been suggested to appear at earlier ages among PWH in comparison to PWoH,^4^ and an overall increased prevalence of CRC among PWH in comparison to PWoH has been observed.^5^ Currently, there are no CRC screening recommendations specifically outlined for PWH. The screening recommendations used for PWoH have been considered sufficient for use among PWH,^6^ despite observations of differences in CRC risk and prevalence between PWH and PWoH. The development of CRC risk prediction models for PWH has the potential to offer personalised screening recommendations.

Comprehensive data including income, civil status, migrant status and comorbidities are collected by national Swedish population-based demographic and health registers. The Swedish National HIV register (InfCareHIV) includes detailed biomedical and demographic data collected from more than 99% of people diagnosed with HIV in Sweden.^7^ This register can be linked with other demographic and health data registers in Sweden to accumulate detailed individual-level data for public health analyses.

This study will use comprehensive nationwide data and apply machine learning (ML) methods to develop a CRC risk prediction model. PWoH will be included to increase statistical power. Matching will be used to control for key confounders, and appropriate methods will be applied to minimise bias and prevent overfitting during model development. Sensitivity and subgroup analyses will be conducted to compare model performance across different scenarios. ML-based prediction models may be superior to logistic or Cox proportional hazards (PH) regression-based models. Previous research studies have compared these approaches, resulting in varying conclusions. Some ML methods have been observed to perform as good as, or may be favourable to, Cox PH or logistic regression for some prediction tasks.^8–12^ Logistic or Cox PH regression methods may perform prediction tasks better or worse than some ML approaches,^11^ but data-driven approaches using ML have been favoured in some studies due to their ability to understand complex relationships in data, which may have an advantageous prediction performance.^12^ It may be more difficult to motivate an ML approach in a resource- or time-limited setting, in particular if data quality or dimensionality is lower and Cox PH or logistic regression methods can complete prediction tasks to a comparable level.

This protocol will use an ensemble method called stacking,^13^ which will combine the predictions of multiple ML models to produce an overall predicted risk score of CRC. Each ML method relies on different statistical assumptions and processes that may reflect different patterns in the data and offer unique insights into the relationships between variables and the outcome.^14^ By leveraging the estimates from each model in the ensemble, the perspectives from diverse model approaches can be integrated to create a more robust and accurate prediction.^15–17^ The performance of the ensemble model approach will be compared with the performance of a Cox PH regression model.

A range of ML methods will be used for the analysis. These algorithms will cluster, classify or identify variables according to their levels of importance in predicting cancer outcomes, aiding in variable selection and prediction model development. Unsupervised ML techniques can explore extensive datasets and examine data without predefined constraints. Supervised ML techniques, however, are still capable of analysing extensive datasets, but in the presence of predefined labels. Using both methods allows for the integration of diverse results from various methodologies, which may strengthen our findings and prediction performance.^16 17^

ML methods and specifically ML prediction modelling have been previously applied to predict certain outcomes for PWH. In clinical care for PWH, ML methods have been applied to predict viral suppression among PWH^18^ and predict who is most at risk of disengaging from ART care.^19^ Similar to the aim of our project, ML methods have been previously applied to develop cancer risk prediction models, but not specifically for PWH.^20 21^ Supervised ML models have been applied to predict the occurrence of cervical precancerous lesions among PWH.^17^ ML methods have also been used to predict comorbidity outcomes as defined by the Charlson Comorbidity Index, which includes cancer, but not as an isolated outcome.^22^ These methods have not yet been applied, to our knowledge, specifically to predict the risk of CRC occurrence among PWH.

In summary, there is a need for updated recommendations and an opportunity to personalise CRC screening recommendations for PWH using robust methods of analysis. This study will aim to develop high-quality risk prediction models by applying various robust ML methods in an ensemble model and comparing it to a baseline Cox PH model. The findings will support model development and provide high-performing tools to help personalise CRC screening recommendations.

### Study aims

The aim of this study is to apply a range of ML methods to analyse comprehensive datasets collected from nationwide demographic and health registers in Sweden to develop CRC risk prediction models for PWH, additionally using data from PWoH and compare the performance to a Cox PH regression model prediction baseline. Additionally, the aim is to evaluate the importance of variables to the prediction models.

## Methods and analysis

### Data sources

Data from the Swedish National HIV Register (InfCareHIV),^7^ the National Cancer Register,^23^ Longitudinal Integration Database for Health Insurance and Labour Market Studies (LISA),^24^ the Total Population Register,^25^ National Cause of Death Register,^26^ National Patient Register^27^ and the National Prescribed Drug Register^28^ will be used in this study.

The Swedish National demographic and health registers, similar to those in other Nordic countries, stand out from the majority of the world through their use and collection of highly detailed nationwide data on an individual level.^29^ A personal identification number is used to identify individuals and record their data in each individual register.^29^ This number can be used to link the registers for research purposes. The linking of the registers is usually performed by Statistics Sweden and the National Board of Health and Welfare. The data delivered usually includes a pseudonymised personal identification number, with the Board keeping the key for identification. Swedish Quality registers are created with the purpose of ensuring equal care development in a specific area of healthcare.^30^ This allows for the collection of highly detailed data relating to specific diseases, conditions, exposures or outcomes of interest. These types of registers are used to monitor rates and diseases, and provide highly detailed data relating to a specified outcome for research.

#### Swedish National HIV Register (InfCareHIV)

The InfCareHIV Register includes data on more than 99% of those diagnosed with HIV in Sweden.^7^ When people are diagnosed or when they immigrate to Sweden with an HIV diagnosis, they are informed that their data will be included in the register but that they may opt-out, and that they may opt-out at any time in the future if they are included. InfCareHIV has collected data from all HIV clinics across Sweden since 2003, and earlier data has been retrospectively added.^7^ The register includes demographic variables, such as legal sex and country of birth, as well as variables relating to HIV acquisition such as date of first positive HIV test and the most likely mode of HIV acquisition. At every follow-up visit, routine measures, such as HIV-RNA levels, CD4(+) T cell counts and current ART, are included in the register.

#### National Cancer Register

The National Cancer Register, managed by the National Board of Health and Welfare, includes all cancer diagnoses in Sweden. Reporting to the register is mandatory by law, and the register includes data on the tumour diagnosis, morphological diagnosis, tumour extent at time of diagnosis, time and place where it was reported, and personal demographic information such as date of birth and place of residence.^23^ The quality of the register is high, with around 99% of the tumours morphologically verified.^31^ The purpose of the register is to follow cancer outcome trends in the population, between subpopulations, as well as contribute to research.

#### National Cause of Death Register

The National Cause of Death Register, managed by the National Board of Health and Welfare, contains reports of all deaths that have occurred in Sweden of residents and non-residents, as well as deaths of residents abroad, including the date and primary cause of death as well as any underlying causes.^26^ When a death occurs, the medical certificate, which is used to determine the underlying cause of death, is filled out by a physician and encompasses a version of the WHO International Form of Medical Certificate of Cause of Death and must be reported within 3 weeks of the event.^26^ The register uses the WHO International Statistical Classification of Diseases and Related Health Problems codes to classify underlying causes of death.^26^ This register additionally acts to monitor causes of death and trends throughout the Swedish population and between subpopulations, as well as contribute to research.

#### National Prescribed Drug Register

The National Prescribed Drug Register, managed by the National Board of Health and Welfare, includes all prescribed drugs distributed in pharmacies in Sweden.^28^ It contains patient, product, prescription, cost and prescriber information.^28^ It includes information on any prescribed drugs dispensed through a pharmacy or on medical devices/consumables.^28^ Trends in prescribed drugs are monitored, and these can additionally be linked to other registers to monitor outcomes.

#### National Patient Register

The National Patient Register, managed by the National Board of Health and Welfare, provides information relating to diseases and specialist care in Sweden.^27^ It includes information on all inpatient stays, outpatient specialised care, compulsory psychiatric admissions and waiting times in emergency departments in Sweden.^27^ Health trends across the country, quality of care and prevention efforts can be monitored with the register.

#### Longitudinal Integration Database for Health Insurance and Labour Market Studies

The LISA Register, managed by Statistics Sweden, collects data on social and economic information on the adult Swedish population. This includes income, main source of income, occupation, sick days, education level and more. The register was initiated to collect data to investigate and address rising levels of sick leave.^24^ The register has been formally collecting data annually since 2003, with data retrospectively until 1990 added by Statistics Sweden using data from the Swedish Public Employment Service, Education Register and the Swedish Social Insurance Agency.^24^ Reporting of this data to the register is compulsory where available and for the general population includes over 95% completeness in income and education reporting.^24^

#### Total Population Register

The Total Population Register, managed by Statistics Sweden, includes every birth, death, emigration from Sweden, immigration to Sweden, migration within Sweden, marriage and divorce, country of birth, legal sex and citizenship.^25^ The primary purpose of this register is to cover the composition and structure of the Swedish population.^25^

### Study population

**Figure 1:**
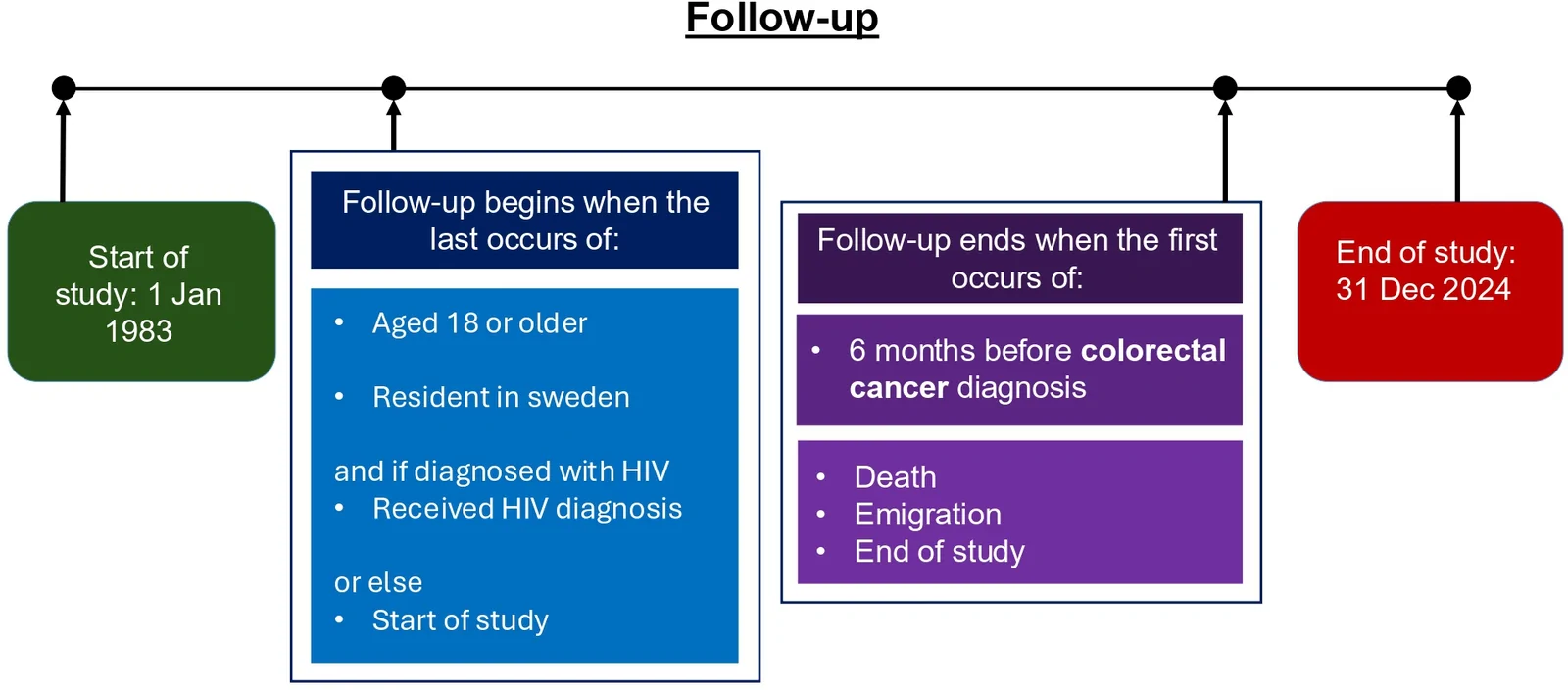
Diagram illustrating study inclusion process.

The study population will include all resident PWH and PWoH, 18 years or older, born between 1940 and 2008, who resided in Sweden at any time point between 1983 and 2024. The study period will span from 1 January 1983 to 31 December 2024. The inclusion date will be the day the person turns 18, the start date of residency in Sweden, or the study start date, whichever occurs last. Follow-up will continue until emigration, death, end of study time or until 6 months before the first incident of CRC. The outcome of interest is CRC. See figure 1 for an illustration of the inclusion criteria and how inclusion and the follow-up period were defined.

### Data analysis plan

A detailed description of the variables of interest under study from each register is provided in online supplemental table 1. Aspects of the study will mirror the protocol applied by Fernández Carbonell and colleagues, and the code available online will be applied throughout.^16^ All data preparation and analysis will be carried out in Stata and Python using Jupyter Notebook in Visual Studio Code.

#### Data preparation

For this study, we will use complete cases only since missing data are minimal. Variables missing more than 10% of the data will be omitted, and after that, any observations with missing data will be dropped.^32^ For the ML analyses, the data will be split 80% into training and 20% into test sets based on follow-up time. The variables in each set will be further prepared to optimise their analysis. Categorical variables will be transformed using one-hot encoding. Normalisation and standardisation of variables will be applied where necessary.

The participants will be matched 1:10 using the entire HIV-negative population in Sweden that fit the criteria. They will be matched on age, legal sex, region of birth and comorbidities including hypertension, chronic obstructive pulmonary disease, diabetes and previous cancer diagnosis.^33 34^ The standardised mean difference of the cohorts will be compared from before and after matching to confirm consistency. Power was calculated using the full anticipated study population consisting of just over 13 000 PWH matched 1:10 to approximately 130 000 PWoH. Median follow-up time is estimated to be at least 10 years in both groups. The expected incidence rate of CRC used for the calculation was the Swedish age-standardised incidence rate reported by the European Commission (129.1 per 100 000 population).^35^ Based on these assumptions and using a two-sided alpha of 0.05, the study is estimated to have 80% power to detect an HR of approximately 1.20 or greater.

#### Cox PH regression

A Cox PH regression model will be used as a baseline performance comparison for the ML methods. This model will favour the simplest approach, and the use of prior knowledge supported by literature will select the most relevant variables. The model performance will then be considered to develop the model for the prediction of cancer among PWH.

#### ML ensemble model—unsupervised ML

Time Series Split cross-validation (CV) will be used during model training and meta-learning stages. K-means and hierarchical clustering methods will be used for the clustering analyses. The clustering analyses will be performed within each CV fold. The optimal number of clusters for the clustering analyses will be determined using both the Calinski-Harabasz Index and Silhouette Scores.^16 36^ The final number of clusters will be selected based on the estimates from these two methods and applied.

The clustering analyses will be used to observe any differences in the clustering patterns across the outcomes.^16 37 38^ These methods can uncover patterns from the data structure without prior information or further input to the analysis.^39^ By interpreting the most decisive variables during the clustering process additionally using decision trees, their importance in predicting the outcome can be evaluated in the process. K-means clustering will be used to group unlabelled data into the pre-estimated number of distinctive clusters depending on their distances from each other.^38^ The algorithm iteratively runs until there are no significant changes to the clusters. Hierarchical clustering groups two observations together which are most similar, and is iteratively updated until the pre-estimated number of distinctive clusters is reached.^37^ Decision trees will be used to aid interpretation of the clustering decisions from both analyses to determine the important contributing variables to the outcome.^16^ If a difference in the clustering patterns over the outcome is observed, we can interpret the most determining variables in the clustering patterns as potentially important to predicting the risk of developing cancer. Cluster labels will be generated from the training data only to avoid data leakage and added as variables to both the training and test sets in all models.

#### ML ensemble model—supervised ML

Elastic Net regularisation, XGBoost, Random Survival Forests and decision trees will be used to develop the ensemble model for predicting CRC risk. For Elastic Net regularisation, a Cox PH model with an Elastic Net penalty will be used to estimate relative risk and interpret variable importance via the penalised regression coefficients. The resulting model additionally highlights influential predictors by minimising less important coefficients towards zero, indicating lower contribution to predicting the outcome.^40^

Decision trees are tree-based models that split data based on variable thresholds to make predictions. Decision trees suited for survival time-to-event data will be used. This method will estimate the probability of cancer over time. This model may additionally be used to infer how much each variable contributes to reducing dataset impurity at each split,^41^ contributing to our understanding of which variables are most valuable to the outcome prediction. Random Survival Forest models work similarly to decision trees but aggregate results across many trees, providing more robust estimates of variable importance and potentially improved performance for risk prediction. XGBoost uses built-in optimisation techniques to develop high-performing prediction models.^42^ The output of the XGBoost model may additionally be used to assess the predictive value of individual variables.

Out-of-fold predictions from each model will be stored to serve as inputs for the meta-learner. Additionally, SHapley Additive exPlanations (SHAP) values will be computed for each trained base learner to assess variable contributions and enhance model transparency.

#### Stacking and dissemination

Stacking, an ensemble learning method, will be used to combine predictions from the previously described ML models.^43^ The base learners will generate out-of-fold risk scores using Time Series Split, which will then serve as inputs to a Cox-PH model as the meta-learner. Finally, the trained base learners and meta-learner will then be used to generate final risk score predictions on the test set.

The performance of the stacked models will be primarily evaluated using balanced accuracy,^44^ and additionally assessed with the concordance index,^45^ Brier score,^46^ time-dependent area under the curve and area under the precision-recall curve (AUPRC), before variable importance is assessed.^13 47 48^ All metrics will be thoroughly considered, but the balanced accuracy of each prediction performance will be weighted most due to its handling of class imbalance, considering both sensitivity and specificity.^44^ AUPRC is preferred over area under the receiver operating curve due to its ability to better handle class imbalance, which will be a factor in this study.^49^

**Figure 2:**
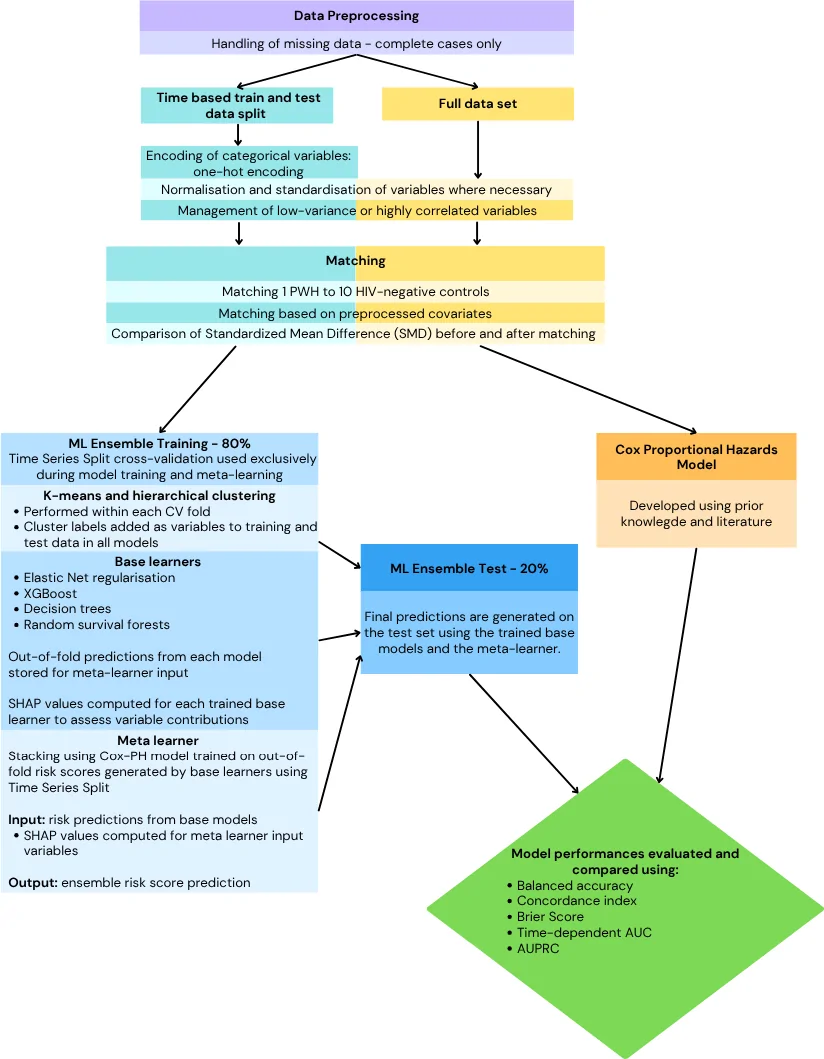
Protocol pipeline. AUC, area under the curve; AUPRC, area under the precision-recall curve; Cox PH, Cox proportional hazards; CV, cross-validation; ML, machine learning; PWH, people with HIV; SHAP, SHapley Additive exPlanations.

The model reporting will adhere to the TRIPOD+AI statement.^50^ The variable importance results will additionally be considered separately. A summary of the analysis plan is illustrated in figure 2.

#### Substudy—PWH only

An analysis will be conducted with PWH only. A simple Cox PH regression model will be developed using preselected covariates using existing knowledge of risk factors. An exploratory approach will be followed, implementing ML methods to handle overfitting and class imbalance as needed. The prediction performance will be assessed using the same methods as the previous models and compared.

#### Sensitivity analysis—incomplete data

A sensitivity analysis, using ML techniques that handle missing data natively, will be conducted with PWH only, and the results will be compared with the substudy results to compare the handling of missing data.

The unsupervised analysis for the incomplete data will consist of principal component analysis and t-distributed stochastic neighbour embedding methods to perform dimensionality reduction. The supervised analysis for the incomplete data will use XGBoost models, decision trees and random forests. All these methods can handle missing values natively and therefore will be implemented for the sensitivity analysis.

## Ethics and dissemination

This study has ethical approval from the Regional Ethical Committee in Sweden (Dnr: 2024-04185-02, 2024-06783-02, 2023-00191-01, 2022-02897-02, 2022-05624-01, 2018/11-31/2). Given that the study is retrospective and register-based, using only pseudonymised data, there are minimal physical, psychological or privacy risks to included individuals. At the time of entering HIV care, PWH are informed about the InfCareHIV registry, after which they can opt-out, which is very rare. They are informed that deidentified data may be used and published for research, given that ethical permission has been granted, and therefore individual consent is not necessary for each individual research project. All results will be presented on population level with no possibility of identification. The long-term risks could include misuse of predictive models if they are not updated with current data or if used to unjustly discriminate against individuals at higher risk. We will advocate for the integration of cancer risk prediction models into routine HIV care, and the ethical ramifications of such new policies will be critically examined. This project is expected to contribute to improved public health outcomes, particularly in CRC prevention and early detection among individuals with HIV. It also aims to inform healthcare policies and potentially reduce healthcare disparities. The beneficiaries of this research include PWH at risk of CRC, healthcare providers and policymakers. The negligible short-term risks are balanced against the substantial long-term value of potentially saving lives through early CRC detection and tailored prevention strategies. Moreover, the research is conducted with patient advocacy and ethical considerations at its core, ensuring that the benefits are maximised while risks are minimised.

### Data storage and security

Pseudonymised register-linkage data are safely stored in a central storage project folder, using Structured Query Language at Karolinska Institutet, Sweden. Only members of the research group can access the data. All data analyses will be performed in the storage folders using a virtual desktop with a two-factor authorisation process. The data will be stored for at least 10 years after the results have been reported and published according to the Swedish Archives Act (1990:782).

### Patient and public involvement

For the planning of this study, we have, since May 2023, involved a patient reference group representing three different HIV patient organisations in Sweden. The reference group is diverse in terms of gender, ethnicity, age and HIV acquisition risk. The research group have regular meetings with the reference group to discuss the aim and design of the study and to ensure that it is aligned with the research questions that the patient organisations have raised as important for the community.

### Clinical relevance

The CRC risk prediction model for PWH will be developed with the aim of supporting risk-stratified CRC surveillance within routine HIV care. The Swedish national CRC screening programme currently invites the general population aged 60–74 years for biennial faecal testing^51^ and does not account for HIV-specific risk factors. A validated risk prediction model could be integrated into the InfCareHIV decision-support interface used at all Swedish HIV clinics, enabling treating physicians at routine annual follow-up to identify individuals who may benefit from earlier screening initiation, shorter screening intervals or referral for colonoscopy. Before clinical deployment, the model will require external validation in independent cohorts and prospective evaluation of its effect on screening uptake, diagnostic yield and stage at diagnosis.

### Dissemination plan

All results will be summarised and presented on a population level in meetings with the involved patient organisations. A manuscript with the results of this study will be produced for publication in a peer-reviewed journal. These findings will further be submitted for consideration for oral and poster presentation at conferences. Findings will also be communicated to the Swedish Reference Group for Antiretroviral Treatment and to the European AIDS Clinical Society to inform future revisions of clinical guidance on cancer prevention in PWH.

### Data availability statement

The individual participant data underlying this article were subject to ethical approval and cannot be shared publicly. Data from the deidentified health registries are not freely available due to protection of the personal integrity of the participants. Data are available from CC on reasonable request.

## Supplementary material

10.1136/bmjopen-2025-108693 online supplemental file 1

## References

[1] Yuan T, Hu Y, Zhou X (2022). Incidence and mortality of non-AIDS-defining cancers among people living with HIV: A systematic review and meta-analysis. EClinicalMedicine.

[2] Zicari S, Sessa L, Cotugno N (2019). Immune Activation, Inflammation, and Non-AIDS Co-Morbidities in HIV-Infected Patients under Long-Term ART. Viruses.

[3] Borges AH, Dubrow R, Silverberg MJ (2014). Factors contributing to risk for cancer among HIV-infected individuals, and evidence that earlier combination antiretroviral therapy will alter this risk. Curr Opin HIV AIDS.

[4] Mills K, Sobukonla T, Bilal M (2022). Young-onset colon cancer among people living with HIV in metropolitan Atlanta. Int J Colorectal Dis.

[5] Mokhtar A, Alameddine Z, Rajavel A (2025). Colorectal cancer risk in the HIV-positive population: A comprehensive analysis. Phys J Med.

[6] O’Neill TJ, Nguemo JD, Tynan A-M (2017). Risk of Colorectal Cancer and Associated Mortality in HIV: A Systematic Review and Meta-Analysis. J Acquir Immune Defic Syndr.

[7] Carlander C, Brännström J, Månsson F (2023). Cohort profile: InfCareHIV, a prospective registry-based cohort study of people with diagnosed HIV in Sweden. BMJ Open.

[8] Tran TT, Lee J, Gunathilake M (2023). A comparison of machine learning models and Cox proportional hazards models regarding their ability to predict the risk of gastrointestinal cancer based on metabolic syndrome and its components. Front Oncol.

[9] Huang RJ, Kwon NS-E, Tomizawa Y (2022). A Comparison of Logistic Regression Against Machine Learning Algorithms for Gastric Cancer Risk Prediction Within Real-World Clinical Data Streams. *JCO Clin Cancer Inform*.

[10] Xu L, Cai L, Zhu Z (2023). Comparison of the cox regression to machine learning in predicting the survival of anaplastic thyroid carcinoma. BMC Endocr Disord.

[11] Bjerre LM, Peixoto C, Alkurd R (2024). Comparing AI/ML approaches and classical regression for predictive modeling using large population health databases: Applications to COVID-19 case prediction. *Glob Epidemiol*.

[12] Sievering AW, Wohlmuth P, Geßler N (2022). Comparison of machine learning methods with logistic regression analysis in creating predictive models for risk of critical in-hospital events in COVID-19 patients on hospital admission. BMC Med Inform Decis Mak.

[13] Kablan R, Miller HA, Suliman S (2023). Evaluation of stacked ensemble model performance to predict clinical outcomes: A COVID-19 study. Int J Med Inform.

[14] Sheng S, Li A, Liu X (2024). Factors and machine learning models for predicting successful discontinuation of continuous renal replacement therapy in critically ill patients with acute kidney injury: a retrospective cohort study based on MIMIC-IV database. BMC Nephrol.

[15] Konar K, Das S, Das S (2025). Employee Attrition Prediction Using Bayesian Optimized Stacked Ensemble Learning and Explainable AI. SN COMPUT SCI.

[16] Fernández Carbonell M, Boman M, Laukka P (2021). Comparing supervised and unsupervised approaches to multimodal emotion recognition. PeerJ Comput Sci.

[17] Namalinzi F, Galadima KR, Nalwanga R (2024). Prediction of precancerous cervical cancer lesions among women living with HIV on antiretroviral therapy in Uganda: a comparison of supervised machine learning algorithms. BMC Womens Health.

[18] Yang X, Cai R, Ma Y (2025). Using Machine Learning Techniques to Predict Viral Suppression Among People With HIV. J Acquir Immune Defic Syndr.

[19] Xie Z, Hu H, Kadota JL (2024). Prevention of adverse HIV treatment outcomes: machine learning to enable proactive support of people at risk of HIV care disengagement in Tanzania. BMJ Open.

[20] Wu X, Tu H, Hu Q (2024). Novel machine learning algorithm in risk prediction model for pan-cancer risk: application in a large prospective cohort. BMJ Oncol.

[21] Tran TT, Lee J, Kim J (2024). Machine learning algorithms that predict the risk of prostate cancer based on metabolic syndrome and sociodemographic characteristics: a prospective cohort study. BMC Public Health.

[22] Yang X, Zhang J, Chen S (2021). Utilizing electronic health record data to understand comorbidity burden among people living with HIV: a machine learning approach. AIDS.

[23] Socialstyrelsen (2024). National cancer register. https://www.socialstyrelsen.se/en/statistics-and-data/registers/national-cancer-register.

[24] Ludvigsson JF, Svedberg P, Olén O (2019). The longitudinal integrated database for health insurance and labour market studies (LISA) and its use in medical research. Eur J Epidemiol.

[25] Ludvigsson JF, Almqvist C, Bonamy A-KE (2016). Registers of the Swedish total population and their use in medical research. Eur J Epidemiol.

[26] Brooke HL, Talbäck M, Hörnblad J (2017). The Swedish cause of death register. Eur J Epidemiol.

[27] Socialstyrelsen (2025). National patient register. https://www.socialstyrelsen.se/en/statistics-and-data/registers/national-patient-register.

[28] Socialstyrelsen (2025). National prescribed drug register. https://www.socialstyrelsen.se/en/statistics-and-data/registers/national-prescribed-drug-register.

[29] Laugesen K, Ludvigsson JF, Schmidt M (2021). Nordic Health Registry-Based Research: A Review of Health Care Systems and Key Registries. Clin Epidemiol.

[30] Emilsson L, Lindahl B, Köster M (2015). Review of 103 Swedish Healthcare Quality Registries. J Intern Med.

[31] Sakakibara S, Pazzagli L, Linder M (2024). Consistency between the National Patient Register and the Swedish Cancer Register. Pharmacoepidemiol Drug Saf.

[32] Mukaka M, White SA, Terlouw DJ (2016). Is using multiple imputation better than complete case analysis for estimating a prevalence (risk) difference in randomized controlled trials when binary outcome observations are missing?. Trials.

[33] Qiu H, Wang L, Zhou L (2023). Comorbidity Patterns in Patients Newly Diagnosed With Colorectal Cancer: Network-Based Study. JMIR Public Health Surveill.

[34] Gheybi K, Buckley E, Vitry A (2022). Occurrence of comorbidity with colorectal cancer and variations by age and stage at diagnosis. Cancer Epidemiol.

[35] Commission E (2026). Colorectal cancer prevention. Knowledge for policy: health promotion and disease prevention knowledge gateway. https://knowledge4policy.ec.europa.eu/health-promotion-knowledge-gateway/colorectal-cancer-1_en.

[36] Ekemeyong Awong LE, Zielinska T (2023). Comparative Analysis of the Clustering Quality in Self-Organizing Maps for Human Posture Classification. *Sensors (Basel*).

[37] Werner E, Clark JN, Hepburn A (2023). Explainable hierarchical clustering for patient subtyping and risk prediction. Exp Biol Med (Maywood).

[38] Nedyalkova M, Madurga S, Simeonov V (2021). Combinatorial K-Means Clustering as a Machine Learning Tool Applied to Diabetes Mellitus Type 2. Int J Environ Res Public Health.

[39] Ramsdale E, Snyder E, Culakova E (2021). An introduction to machine learning for clinicians: How can machine learning augment knowledge in geriatric oncology?. J Geriatr Oncol.

[40] Hong F, Tian L, Devanarayan V (2023). Improving the Robustness of Variable Selection and Predictive Performance of Regularized Generalized Linear Models and Cox Proportional Hazard Models. Mathematics (Basel).

[41] Hu L, Li L (2022). Using Tree-Based Machine Learning for Health Studies: Literature Review and Case Series. Int J Environ Res Public Health.

[42] Tarwidi D, Pudjaprasetya SR, Adytia D (2023). An optimized XGBoost-based machine learning method for predicting wave run-up on a sloping beach. MethodsX.

[43] Mahajan P, Uddin S, Hajati F (2023). Ensemble Learning for Disease Prediction: A Review. Health Care (Don Mills).

[44] Owusu-Adjei M, Ben Hayfron-Acquah J, Frimpong T (2023). Imbalanced class distribution and performance evaluation metrics: A systematic review of prediction accuracy for determining model performance in healthcare systems. *PLOS Digit Health*.

[45] Brentnall AR, Cuzick J (2018). Use of the concordance index for predictors of censored survival data. Stat Methods Med Res.

[46] Stehouwer N, Rowland-Seymour A, Gruppen L (2025). Validity and reliability of Brier scoring for assessment of probabilistic diagnostic reasoning. Diagnosis (Berl).

[47] Cabot JH, Ross EG (2023). Evaluating prediction model performance. Surgery.

[48] Hicks SA, Strümke I, Thambawita V (2022). On evaluation metrics for medical applications of artificial intelligence. Sci Rep.

[49] Saito T, Rehmsmeier M (2015). The Precision-Recall Plot Is More Informative than the ROC Plot When Evaluating Binary Classifiers on Imbalanced Datasets. PLoS ONE.

[50] (2024). TRIPOD+AI statement: updated guidance for reporting clinical prediction models that use regression or machine learning methods. BMJ.

[51] Jørgensen SF, Njor SH, Nevala A (2025). Nordic colorectal cancer screening programmes: A comparison of organization, operation, and quality indicators. Eur J Cancer.

