# Supplementary material for: Comparison of an ensemble machine learning model to a Cox regression model to predict colorectal cancer risk among people with HIV using retrospective nationwide cohort data in Sweden: a study protocol
Source: BMJ Open. 2026 Sep 9;16(9):e108693. doi: 10.1136/bmjopen-2025-108693 (PMC13560964; doi:10.1136/bmjopen-2025-108693)
Supplement: Supplementary file 1 [file bmjopen-2025-108693.reviewer_comments.pdf]

PEER REVIEW HISTORY

BMJ Open publishes all reviews undertaken for accepted manuscripts. Reviewers are asked to complete a checklist review form and are provided with free text boxes to elaborate on their assessment. These free text comments are reproduced below.

ARTICLE DETAILS

Title (Provisional)

Comparison of ensemble machine learning model to Cox regression model to predict colorectal cancer risk among people with HIV using retrospective nationwide cohort data in Sweden: a study protocol

Authors

Nilsson, Josefin; Deng, Yunyang; Elvstam, Olof; Killander-Möller, Isabela; Lei, Jiayao; Mansson, Fredrik; Naucler, Pontus; Nygren, Jonas; Ruhe-van der Werff, Suzanne; Wagner, Philippe; Yilmaz, Aylin; Brännström, Johanna; Boman, Magnus; Carlander, Christina

---

VERSION 1 - REVIEW

**Reviewer**	**1**
**Name**	**Leopa , Nicoleta**
**Affiliation**	**Ovidius University of Constanta**
**Date**	**20-Apr-2026**
**COI**	**None**

This manuscript presents a well-designed and clearly structured study addressing an important and timely topic. The objectives are clearly stated, the methodology is appropriate, and the results are presented in a coherent and logical manner. The manuscript is generally well written and contributes meaningful data to the field.

The study's strengths include the clear analytical framework and the effort to contextualize findings within existing literature. The overall scientific quality supports publication.

A few minor revisions would further strengthen the manuscript:

Clarification of clinical implications

While the findings are clearly presented, their direct impact on clinical practice could be more explicitly stated. For example, in the Discussion, the authors may consider adding a short paragraph outlining how these results could influence patient management, risk stratification, or decision-making pathways.

Expansion of limitations

The limitations section could be slightly expanded to address potential residual confounding and generalizability. For instance, if the study is single-center or based on a specific population, this should be explicitly acknowledged as a factor that may limit external applicability.

Moderation of causal language

Some statements in the Discussion appear slightly overstated. For example, phrases such as "this study demonstrates that…" could be revised to "this study suggests that…" to better reflect the observational nature of the data.

Minor clarity and consistency issues

In the Methods section, the description of inclusion criteria could be slightly clarified to improve reproducibility.

In the Results section, ensure consistency between numerical values reported in the text and those presented in tables (even small discrepancies may affect clarity).

In the Discussion, a few longer sentences could be simplified to improve readability.

Overall, this is a solid and relevant study with good methodological rigor. I recommend acceptance after minor revision.

VERSION 1 - AUTHOR RESPONSE

Reviewer(s)' Comments to Author
Reviewer: 1
Dr. Nicoleta Leopa , Ovidius University of Constanta
Comments to the Author:

This manuscript presents a well-designed and clearly structured study addressing an important and timely topic. The objectives are clearly stated, the methodology is appropriate, and the results are presented in a coherent and logical manner. The manuscript is generally well written and contributes meaningful data to the field.
The study's strengths include the clear analytical framework and the effort to contextualize findings within existing literature. The overall scientific quality supports publication.

Author response:
We thank you for this supportive summary.

A few minor revisions would further strengthen the manuscript:
Clarification of clinical implications
While the findings are clearly presented, their direct impact on clinical practice could be more explicitly stated. For example, in the Discussion, the authors may consider adding a short paragraph outlining how these results could influence patient management, risk stratification, or decision-making pathways.

Author response:
Thank you for this important comment. This has been elaborated on and clarified under Clinical relevance on page 9 lines 601-610.

Expansion of limitations
The limitations section could be slightly expanded to address potential residual confounding and generalizability. For instance, if the study is single-center or based on a specific population, this should be explicitly acknowledged as a factor that may limit external applicability.

Author response:
Thank you for this relevant comment. All people diagnosed with HIV in Sweden will be included in this study and therefore this study is not limited to a single-center or sub-population. Residual confounding and generalisability has been clarified in the limitations section to better reflect these central issues on page 2 lines 172-175 and 179-182.

Moderation of causal language
Some statements in the Discussion appear slightly overstated. For example, phrases such as "this study demonstrates that..." could be revised to "this study suggests that..." to better reflect the observational nature of the data.

Author response:
Thank you for this very valid comment. The use of any causal language was reviewed and revised accordingly. Changes were made on page 4 line 273, page 9 lines 601-610.

Minor clarity and consistency issues
In the Methods section, the description of inclusion criteria could be slightly clarified to improve reproducibility.

Author response:
Thank you for this comment. The inclusion criteria were clarified in the methods secion on page 6 lines 402-405. Figure 1 was additionally clarified accordingly.

In the Results section, ensure consistency between numerical values reported in the text and those presented in tables (even small discrepancies may affect clarity).

Author response:
Thank you for this comment. Any discrepancies between numbers or terminology presented in Figure 1, Table 1 and the text have been revised.

In the Discussion, a few longer sentences could be simplified to improve readability.

Author response:
Thank you for this comment. The ethics and dissemination portion of the protocol was revised and simplified to improve readability on page 9 lines 601-610.

Overall, this is a solid and relevant study with good methodological rigor. I recommend acceptance after minor revision.

Author response:
We thank the reviewer for this very positive conclusion.
