# Supplementary material for: Comparison of an ensemble machine learning model to a Cox regression model to predict colorectal cancer risk among people with HIV using retrospective nationwide cohort data in Sweden: a study protocol
Source: BMJ Open. 2026 Sep 9;16(9):e108693. doi: 10.1136/bmjopen-2025-108693 (PMC13560964; doi:10.1136/bmjopen-2025-108693)
Supplement: Supplementary file 2 [file bmjopen-16-9-s001.docx]

**Supplementary table 1: Description of variables included from the listed national registers in Sweden**

| **Variable** | **Description** | **Scale/Categorisation** |
| --- | --- | --- |
| **Swedish National HIV Register (InfCareHIV)** | | |
| Sex | Legal sex | Female  Male |
| Gender | Gender identity | Woman  Man  Non-Binary  Transman  Transwoman |
| Region of birth | Geographical region of birth | Asia and Pacific  Eastern Europe and Central asia  Latin America and the Carribean  Middle East and North Africa  Sub-Saharan Africa  Sweden  Western Europe except Sweden |
| Acquisition mode | Most likely mode in which HIV acquisition occurred | Sex with opposite sex  Men who have sex with men  Injection Drug Use (IDU)  Mother to child  Blood transfusion  Unknown |
| First **HIV** ever recorded |  | Positive HIV-serology or HIV-RNA (Copies/mL) |
| Date first **HIV** ever recorded |  |  |
| Last ever recorded **HIV** |  | Copies/mL |
| Date last ever recorded **HIV** |  |  |
| Highest ever recorded **HIV** |  | Copies/mL |
| Date highest ever recorded **HIV** |  |  |
| First ever recorded **CD4** |  | Cells/μL |
| Date first ever recorded **CD4** |  |  |
| Last ever recorded **CD4** |  | Cells/μL |
| Date last ever recorded **CD4** |  |  |
| Nadir **CD4** |  | Cells/μL |
| Date nadir **CD4** |  |  |
| First CD4:CD8 |  | Cells/μL in ratio |
| Last CD4:CD8 |  | Cells/μL in ratio |
| Lowest CD4:CD8 |  | Cells/μL in ratio |
| First ART date |  |  |
| Proportion of observation time with suppressed **HIV RNA** |  |  |
| Proportion of observation time with low **CD4** |  |  |
| Time on individual antiretroviral therapy drug during observation  (n=37) | One variable for each drug. Variable contains a count of time during observation in which the individual drug was taken | Treatment time in days for each of: Abacavir, Zalcitabin, Didanosin, Emtricitabin, Lamivudin, Stavudin, Tenofovir (TDF), Zidovudine, Efavirenz, Nevirapin, Atazanavir, Fosamprenavir, Indinavir, Lopinavir, Nelfinavir, Ritonavir, Saquinavir, Tipranavir, Enfuvirtid, Ritonavir (Boostrare), Lamivudin, Darunavir, Etravirin, Raltegravir, Maravirok, Rilpivirine, Elvitegravir, Cobicistat, Dolutegravir, Tenofovir (TAF), Cabotegravir, Cabotegravir LA, Rilpivirine LA, Bictegravir, Doravirin, Ibalizumab and/or Fostemsavir |
| Time on any integrase inhibitor (INSTI) antiretroviral therapy anchor drugs during observation | Time during observation on any INSTI | Days |
| Time on any non-nucleoside reverse transcriptase inhibitors (NNRTI) antiretroviral therapy anchor drugs during observation | Time during observation on any NNRTI | Days |
| Time on protease inhibitor (PI) antiretroviral therapy anchor drugs during observation | Time during observation on any PI | Days |
| Total drug time | Total time count during observation taking any antiretroviral therapy related drugs | Days |
| **Longitudinal integration database for health insurance and labour market studies (LISA)** | | |
| Highest income recorded over observation time |  |  |
| Average income over observation time |  |  |
| Highest ever recorded education level |  |  |
| Cumulative days unemployed |  |  |
| **National Prescribed Drug Register** | | |
| Comorbidity drugs taken during observation (n=6) | One variable for each drug category. Variable contains a count of time in days during observation in which the individual drug relating to a particular comorbidity | Indication for any drugs relating to: diabetes, obstructive lung disease, hyperlipidaemia, cardiac disease, immune disorders and/or hypertension |
| **National Cancer Register** | | |
| Cancer indicator | Yes or no cancer outcome |  |
| Cancer type |  |  |
| Date of cancer diagnosis |  |  |
| **National Death Register** | | |
| Dead indicator |  |  |
| Date of death |  |  |
| **National Patient Register** | | |
| Hypertension indicator |  |  |
| Date of hypertension occurrence |  |  |
| Diabetes indicator |  |  |
| Date of diabetes diagnosis |  |  |
| Chronic obstructive pulmonary disease indicator |  |  |
| Date of chronic obstructive pulmonary disease occurrence |  |  |
| **Total Population Register** | | |
| Birth year |  |  |
| Sex |  |  |
| Date of migration |  |  |
| Immigration or emigration |  |  |
